# Multiscale Investigation of Modified Recycled Aggregate Concrete on Sulfate Attack Resistance

**DOI:** 10.3390/ma18071450

**Published:** 2025-03-25

**Authors:** Xue-Fei Chen, Xiu-Cheng Zhang, Guo-Hui Yan

**Affiliations:** 1 School of Civil Engineering, Putian University, Putian 351100, China; 2 Engineering Research Center of Disaster Prevention and Mitigation of Southeast Coastal Engineering Structures (JDGC03), Fujian Province University, Putian 351100, China; 3 Jinxi Holding Group Co., Ltd., Putian 351100, China

**Keywords:** construction waste, concrete, recycled aggregates

## Abstract

This study investigated the sulfate resistance of modified recycled aggregate concrete (RAC) by applying carbonation and nano-silica soaking methodologies. Recycled concrete aggregates (RCA) derived from concretes of C30 and C60 strength grades were subjected to these modification techniques and subsequently utilized in the fabrication of RAC specimens. The results show notable porosity and crack density within the interfacial transition zone (ITZ) interfacing recycled aggregate and cement paste in recycled aggregate concrete (RAC). Specifically, the porosity within the ITZ of RAC is observed to be up to 30% higher than that of virgin aggregate concrete. These pathways facilitate the penetration of sulfate ions, subsequently inducing deterioration and resulting in a compression strength reduction of up to 40%. While carbonation treatment exhibits a moderate enhancement in sulfate resistance, decreasing the sulfate penetration depth by 15%, the incorporation of 2% nano-silica by weight of cement proves significantly more effective. This addition reduces the sulfate penetration depth by over 30% and lowers the sulfate concentration by 25%. Furthermore, the compressive strength of RAC modified with nano-silica increases by 15% following 28 days of sulfate exposure. Additionally, a 30% reduction in the sulfate ion mass equilibrium depth is observed in nano-silica-modified RAC, accompanied by a markedly lower sulfate concentration in the pore solution. After 56 days of sulfate attack, the compressive strength of nano-silica-modified RAC retains 85% of its initial value, whereas unmodified RAC decreases to 70%. Notably, the quality of recycled aggregate significantly impacts sulfate resistance, with high-strength RCA (exceeding 40 MPa) demonstrating superior resistance compared to low-strength RCA (below 20 MPa). Consequently, RAC produced with high-strength RCA experiences only a 20% loss in compressive strength under sulfate attack, whereas RAC containing low-strength RCA suffers a 40% loss. The novelty of this study is the effective use of nano-silica soaking and carbonation to enhance the sulfate resistance and compressive strength of recycled aggregate concrete originated from both normal and high-strength reference concrete.

## 1. Introduction

Concrete, as a building material, holds an unparalleled position in terms of widespread application. Its annual production in China alone surpasses 3 billion cubic meters, with the increase rate c.a. 5% per year [1]. However, amidst the rapid socio-economic evolution, its utilization is increasingly encountering multifaceted challenges [2,3,4]. On the one axis, the substantial solid waste generated from the demolition of obsolete structures poses acute environmental concerns. Conversely, the national “dual-carbon” targets of China have placed the construction industry at the crossroads of rapid development and the imperative for energy conservation and emission reduction [5]. Consequently, the recycling of waste aggregates has emerged as a pivotal factor for the sustainable progression of the construction sector.

Nonetheless, recycled aggregate concretes (RACs) often exhibit suboptimal mechanical properties and durability owing to their inherent multitude of defects [6,7]. The incorporation of recycled powder by 30% will reduce the compressive strength of the end product by 11%, as was reported by [8,9]; while the chopped basalt fiber was proved to mitigate this kind of strength loss [10,11,12]. These shortcomings can significantly hinder their widespread adoption and application in the construction industry, despite the pressing need for sustainable materials. It is widely acknowledged that old mortar adhering to the surface of recycled coarse aggregates (RCAs) constitutes a primary source of these deficiencies [13,14]. Understanding the mechanisms behind these deficiencies is crucial for developing effective strategies to improve the performance of RACs [15]. The old mortar adhering to RCAs introduces several challenges. Firstly, this mortar typically has a lower strength and higher porosity compared to fresh mortar. When incorporated into new concrete mixes, these properties can negatively affect the overall strength and durability of the RAC. The aged mortar often contains cracks, voids, and other imperfections that can act as stress concentrators, leading to premature failure under load.

Moreover, the formation of multiple interface transition zones (ITZs) between aggregates and cement mortar within concrete can be attributed to a phenomenon known as the barrier effect [16]. The ITZ is a thin layer of cementitious material that surrounds the aggregate particles. It plays a critical role in the mechanical behavior and durability of concrete. In RACs, the presence of old mortar adhering to RCAs exacerbates this barrier effect. The old mortar acts as a physical barrier, preventing cement particles from effectively filling the voids in the vicinity of the aggregate surface [16,17,18]. This results in a weaker bond between the aggregate and the surrounding mortar, further compromising the mechanical properties of the RAC. The broadening of ITZs due to the old mortar adhering to RCAs leads to a more porous and less cohesive microstructure within the RAC [19,20]. These larger ITZs are more susceptible to the ingress of water, chloride ions, and other harmful substances, which can accelerate the deterioration process. Wu et al. [21] found that concrete with a 100% recycled aggregate replacement rate retained only 70.3% of its compressive strength after exposure to 400 °C and 15 freeze–thaw cycles, compared to concrete with no replacement. Liu et al. [22]. found that recycled concrete performed similarly to conventional concrete under low freeze–thaw cycles, but cracks appeared earlier, and the performance gap widened as cycles exceeded 50%. Deng et al. [23] found that adding air-entraining agents improved the internal pore structure of recycled concrete, with 50% recycled aggregate substitution showing the most stable performance. Consequently, RACs with broadened ITZs exhibit reduced resistance to freeze–thaw cycles, chemical attack, and other durability-related issues.

Given these challenges, the pretreatment of RCAs to enhance their properties assumes paramount importance. Pretreatment methods aim to remove or modify the old mortar adhering to the RCA surface, thereby reducing the adverse effects on the mechanical properties and durability of RACs [20]. Various pretreatment techniques have been explored in the literature, each with its own set of advantages and limitations. Mechanical methods, such as crushing and sieving, can be used to physically remove the old mortar from the RCA surface. While these methods are relatively simple and low-cost, they may not be effective in completely removing the mortar, especially in cases where the mortar is strongly adhered to the aggregate [18]. Additionally, mechanical treatment can lead to the generation of fine particles, which may need to be separated and disposed of properly. Thermal methods involve heating the RCAs to high temperatures to burn off the aged mortar. This approach can be effective in removing a significant portion of the aged mortar, but it may also alter the physical and chemical properties of the aggregate itself [24]. High temperatures can cause thermal cracking and decomposition of the aggregate minerals, leading to a decrease in strength and durability. Chemical methods, such as acid washing, involve treating the RCAs with chemical solutions to dissolve or weaken the bond between the aged mortar and the aggregate. These methods can be more selective in removing the aged mortar while preserving the integrity of the aggregate. However, they may require the use of hazardous chemicals and generate waste solutions that need to be disposed of safely [25]. More recently, researchers have explored the use of microbial-induced calcite precipitation (MICP) as a pretreatment method for RCAs [26,27,28,29]. This technique involves the use of bacteria that produce urease, an enzyme that catalyzes the hydrolysis of urea into ammonia and carbon dioxide [30]. The ammonia raises the pH of the surrounding environment, promoting the precipitation of calcium carbonate. This calcium carbonate can fill in the voids and cracks in the aged mortar, effectively sealing the surface of the RCA. MICP offers several advantages, including the use of environmentally friendly materials and the potential for in situ treatment. However, it requires careful control of the bacterial cultures and precipitation conditions to achieve optimal results.

In addition to pretreatment methods, researchers have also investigated the use of admixtures and supplementary cementitious materials (SCMs) to improve the properties of RACs [31]. Admixtures, such as superplasticizers and viscosity-modifying agents, can enhance the workability and durability of RACs by modifying the rheological properties of the mix. SCMs, such as fly ash, slag, and silica fume, can react with calcium hydroxide produced during cement hydration to form additional cementitious compounds, thereby improving the strength and durability of the RAC [32,33]. While recycled aggregate concretes offer significant environmental benefits, their suboptimal mechanical properties and durability pose significant challenges. The aged mortar adhering to the surface of recycled coarse aggregates is a primary source of these deficiencies, exacerbating the barrier effect and broadening the interface transition zones within the concrete. Pretreatment of RCAs through mechanical, thermal, chemical, or biological methods can help mitigate these adverse effects. Additionally, using admixtures and supplementary cementitious materials can further enhance the properties of RACs. By continuing to explore and refine these strategies, the construction industry can move closer to achieving more sustainable and resilient concrete materials.

Currently, RCA pretreatment methodologies can be broadly delineated into two primary categories: one involves the physical or chemical removal of aged mortar adhering to the RCA surface, encompassing techniques such as mechanical grinding, ultrasonic treatment, and soaking in acidic solutions, among others. However, these methodologies are often fraught with drawbacks, including high energy consumption, environmental pollution, and the potential introduction of erosive substances in the case of acidic solution soaking. An alternative, commonly employed approach entails the reinforcement of the mortar adhering to the RCA surface. Among the various reinforcement techniques, accelerated carbonation technology has garnered considerable research attention. This technology leverages the reaction between CO_2_ and cement hydration products to generate insoluble CaCO_3_, which serves to fill the pores between mortars [34]. Additionally, microbial mineralization deposition technology has been explored by some scholars as a means to achieve similar modification objectives.

More recently, researchers have proposed the utilization of highly reactive nanomaterials, such as nano-SiO_2_ [1,35,36,37,38], for the reinforcement of RCAs. In light of these advancements, the present study undertakes the modification and repair of RCAs sourced from two distinct strength grades of concrete using accelerated carbonation and soaking in nano-silica solutions, respectively. The objective is to prepare different modified RACs and investigate their altered mechanical properties and durability characteristics under sulfate attack conditions. By doing so, this study aims to provide valuable insights and guidance for the formulation of high-performance RACs, thereby contributing to the advancement of sustainable construction practices.

## 2. Experimental Details

### 2.1. Materials

The RCAs (5–10 mm) were sourced from parent concretes with designated compressive strengths of C30 and C60, respectively. These concrete underwent standard curing protocols for 180 days, adhering to industry standards. Subsequently, they were processed through a jaw crusher to achieve particle fragmentation and were then subjected to a meticulous screening process to attain a uniform particle size distribution within the specified range of 5 to 27 mm, henceforth designated as J-RCA. The cement employed was of grade P·O 42.5 (Huarun, Shenzhen, China) categorized as ordinary Portland cement. The natural coarse aggregate consisted of granite crushed stone, while the natural fine aggregate comprised river sand. Tap water served as the mixing medium, and a polycarboxylate-based superplasticizer was incorporated to enhance the workability and flow properties of the concrete mixture.

Following the guidelines outlined in the Specification for Mix Proportion Design of Ordinary Concrete. The mix proportions for the reference concretes were calculated and validated. The resultant mix proportions were then used to produce the reference concretes, which were subsequently tested for their 28-day cubic compressive strengths. The test results, presented in Table 1, demonstrate the high quality and consistency of the reference concretes, thereby affirming the reliability of the RCAs derived from them.

### 2.2. Sample Preparation and the Sulfate Attack

**Figure 1:**
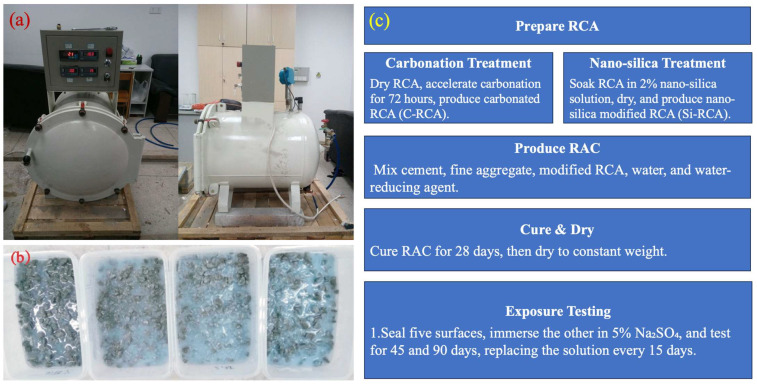
In-site graphs and the flow chart of the sample preparation process: (**a**) accelerated carbonation; (**b**) nano-silica soaking; (**c**) flow chart.

The prepared RCAs were treated by the carbonation and nano-silica soaking method (see Figure 1). Regarding the carbonation treatment procedure, RCAs were firstly dried to attain constant weight and then underwent a 72 h accelerated carbonation process within a lab-made carbonation chamber. This process results in the production of carbonation-modified recycled coarse aggregates, specifically designated as C-RCA30 and C-RCA60, where the numerical suffixes denote their respective reference concrete strength grades. On the contrary, the modification with nano-SiO_2_ via the immersion technique was also employed. The RCA was fully submerged in a nano-SiO_2_ solution with a concentration of 2%. Following complete immersion, the RCA is subsequently retrieved and subjected to drying in the oven for 12 h to ensure thorough evaporation of any residual moisture. The process yields nano-SiO_2_-modified recycled coarse aggregates, designated as Si-RCA30 and Si-RCA60, respectively, again with the numerical suffixes reflecting their corresponding reference concrete strength classifications.

The prepared RCAs, including carbonation-modified and nano-SiO_2_-modified ones, were then used to produce the RAC as per the following proportion: cement, fine aggregate, carbonation- or nano-silica-modified recycled coarse aggregate, water, and water-reducing agent being controlled at 340 kg/m^3^, 740 kg/m^3^, 970 kg/m^3^, 163 kg/m^3^, and 3.2 kg/m^3^, respectively. Following a period of standard curing for 28 days and subsequent drying to constant weight, the RAC specimens underwent further preparation for exposure testing. Five surfaces of each specimen were sealed with paraffin wax to prevent ingress of the test solution, while the one remaining surface was fully immersed in a Na_2_SO_4_ solution of a precisely determined mass fraction of 5%. This immersion was conducted for durations of 45 and 90 days, respectively, to evaluate the long-term durability of the RAC specimens under sulfate attack. To maintain the consistency of the test conditions and ensure accurate results, the Na_2_SO_4_ solution was replaced every 15 days throughout the immersion period.

### 2.3. Testing

#### 2.3.1. Morphological and Compositional Examination of RCA

The surface morphology and composition of the modified RCA were investigated. Scanning Electron Microscopy (SEM; model JSM-7610F, JEOL Ltd., Tokyo, Japan) was employed to observe and analyze the surface characteristics of the modified RCA. Additionally, X-ray Powder Diffraction (XRD; D8 Advance X, Bruker, Billerica, MA, USA) was utilized to determine the composition of the substances present on the surface of the modified RCA.

#### 2.3.2. Compressive Strength Evaluation and Sulfate Concentration Assessment of RAC

The compressive strength of RAC subjected to various sulfate attack durations was tested in accordance with the Standard Test Method for Mechanical Properties of Ordinary Concrete. Furthermore, the sulfate ion concentration within RAC at different attack durations was quantified using spectrophotometry [39]. The sulfate-attacked RAC samples were milled into fine powder and dissolved in distilled water. Subsequent filtration was followed by the addition of a mixture of hydrochloric acid (2.5 mol/L) and barium chloride to obtain the analyte solution. For quantitative analysis of the sulfate ions in the analyte solution, a standard solution of sodium sulfate (Na_2_SO_4_) was prepared to establish a standard absorbance curve and corresponding fitting equation, with absorbance denoted as A.

#### 2.3.3. Micro-Mechanical Testing of RAC

The micro-mechanical properties of the interfacial transition zones (ITZs) within RAC were analyzed using a microhardness tester (HX-1000T, Sanyi, Guangzhou, China).

(1) Specimen Fabrication: A thin section with a thickness of 10 mm was precisely cut perpendicular to the erosion surface of the RAC using a concrete cutting machine. This thin section was then further divided into two specimens, each with dimensions of 100 mm × 50 mm × 10 mm, using a metallographic cutting machine. The specimens were subsequently dried to constant weight in an oven at 50 °C after replacing the free water with anhydrous ethanol. The specimens underwent grinding using a metallographic grinding machine, employing sandpaper with grit sizes ranging from 75 to 10 μm, while anhydrous ethanol served as a coolant and lubricant. Ultimately, the specimens were polished to produce samples suitable for microhardness testing.
(2) Testing Protocol: Within the vicinity of a single aggregate, three types of ITZs were identified: old aggregate-old mortar (LG-LS), old aggregate-new mortar (LG-XS), and old mortar-new mortar (LS-XS). Nine lattices, each containing a 4 × 5 grid of measurement points, were tested within each ITZ. To prevent overlapping of adjacent indentations, the test load was carefully calibrated to 50 g. Also, the longitudinal distance (L_1_) and horizontal distance (L_2_) between adjacent indentations were both equivalent to the vertical height difference (h) of 10 μm between transversely adjacent indentations. Notably, the first indentation of each lattice, located at a distance of 0 μm from the ITZ boundary, was positioned directly on the interfacial boundary.

#### 2.3.4. Thermogravimetric of RAC

To further elucidate the composition of hydrated products within RAC after 90 days of sulfate attack, thermogravimetric analysis (TGA; SDTQ600) was conducted.

## 3. Results and Discussion

### 3.1. Morphological Graph and Composition of RCA

**Figure 2:**
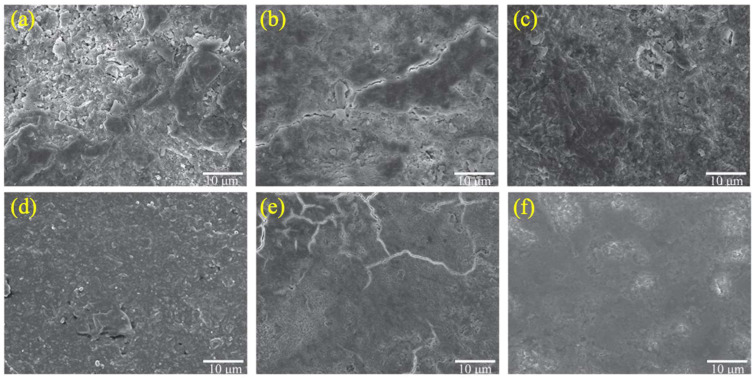
Micrographs of modified and original RCAs: (**a**) J-RCA-30; (**b**) C-RCA-30; (**c**) Si-RCA-30; (**d**) J-RCA-60; (**e**) C-RCA-60; (**f**) Si-RCA-60.

Upon closer examination of Figure 2a, it becomes apparent that the surface of RCA30, subsequent to simple crushing, is adorned with a relatively porous and loosely adhered layer of old mortar. This layer is characterized by an abundance of pores and cracks, which compromise its structural integrity. Upon undergoing carbonation treatment, however, the surface of C-RCA30’s old mortar undergoes a marked reduction in both the number and size of pores. Nonetheless, it is still discernible that there persist notable crack interfaces and transition zones across its surface. When subjected to immersion in a solution of nano-SiO_2_, the old mortar’s surface transforms, with pores becoming less prevalent, and the overall structure becomes more compact. This compaction is accompanied by an enrichment of hydration products that are more intricately interconnected. This transformation is attributable to the pozzolanic and filling effects of nano-SiO_2_, which are well-documented phenomena. Figure 2d–f offer further insights into the microstructural characteristics of RCA. They reveal that in comparison to RCA30, RCA derived from high-strength concrete exhibits a surface that is dense and smooth. This is particularly evident in the nano-SiO_2_-modified samples, where the old mortar assumes an even more flattened and densified morphology.

**Figure 3:**
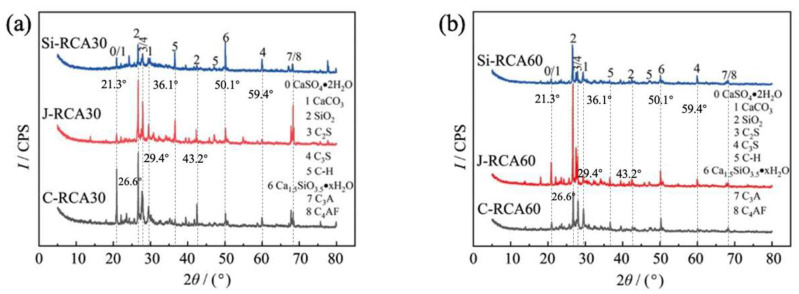
XRD pattern of modified and original RCAs: (**a**) RCA30 group; (**b**) RCA60 group.

Turning our attention to Figure 3, which presents the results of XRD analysis, it can be seen that the surface mortar of J-RCA30 is laden with incompletely hydrated cement clinker. This is a contributing factor to its porous and loose surface texture. After undergoing carbonation treatment, there is a notable increase in the content of CaCO_3_. This increase is indicative of the reaction between CO_2_ and Ca(OH)_2_ within the old mortar, resulting in the formation of CaCO_3_. Following modification with nano-SiO_2_, there is a marked decrease in the amount of incompletely hydrated cement clinker. Concurrently, there is a significant increase in the content of Ca_15_SiO_3.5_·*x*H_2_O. This suggests that nano-silica not only catalyzes the further hydration of cement clinker but also reacts with hydration products to form a gel. This gel subsequently undergoes crystallization, leading to an augmentation in the content of Ca_15_SiO_3.5_·*x*H_2_O. This result is consonant with the observed compact and smooth surface morphology. When comparing RCA30 to RCA60, as depicted in Figure 3b, it becomes evident that the latter contains a markedly reduced amount of incompletely hydrated cement clinker. This suggests that the cement hydration process in C60 is more complete, which translates into a higher degree of density (as evidenced in Figure 2d–f). This enhanced density is likely a contributing factor to the improved mechanical properties and durability of RCA60, making it a more favorable material for use in various construction applications.

### 3.2. Compressive Strength and Sulfate Erosion Concentration Profiles in RAC

**Figure 4:**
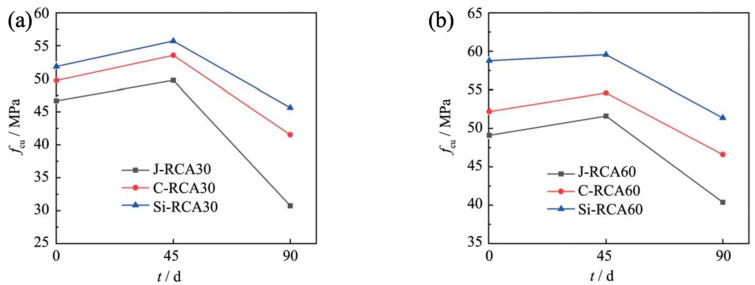
Compressive strength of recycled aggregate concrete (RAC) with various sulfate erosion periods: (**a**) RCA30 group; (**b**) RCA60 group.

The study presents a detailed examination of the compressive strength and sulfate erosion concentration in RAC, with particular emphasis on the effects of modifications such as nano-SiO_2_ incorporation and carbonation treatment. Figure 4 depicts the compressive strength results of RAC. Notably, the compressive strength values of modified RAC exceed those of unmodified RAC, with nano-SiO_2_ modification exhibiting a more pronounced enhancement compared to carbonation treatment. Subsequent to sulfate attack, all samples exhibited an increase in compressive strength to varying degrees. This phenomenon can be attributed to the reaction between aluminum-containing hydrated products within the cementitious matrix and infiltrating sulfate ions, which results in the formation of expansive ettringite (AFt). The AFt fills the pores and cracks within the concrete matrix, thereby enhancing the mechanical properties of RAC. However, as the erosion duration extended to 90 days, the generation of a substantial quantity of expansive hydrated products, such as AFt, disrupted the microstructure of RAC, leading to a marked decline in compressive strength. This decrement was particularly pronounced in unmodified RAC, with a reduction of 31.7% and 22.1%, respectively. Notably, nano-silica modification and carbonation treatment were found to effectively mitigate the adverse effects of sulfate erosion.

**Figure 5:**
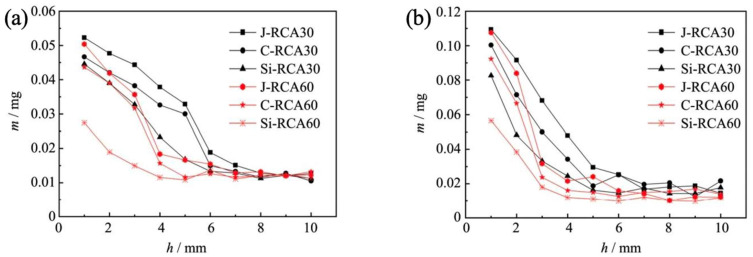
Sulfate penetration depth of recycled aggregate concrete (RAC): (**a**) RCA30 group; (**b**) RCA60 group.

Figure 5 illustrates the sulfate penetration depth results, offering insights into the sulfate ion mass distribution within different RAC samples. At a duration of 45 days, it is evident that as the penetration depth increases, the sulfate ion mass decreases progressively in layers within the RAC samples. Specifically, for RCA30, the sulfate ion mass stabilizes at a penetration depth of approximately 9 mm, whereas C-RCA60 and Si-RCA60 reach equilibrium at depths of 5 mm and 6 mm, respectively. Furthermore, at any given depth, the sulfate mass in modified RAC is notably lower than that in unmodified RAC.

Upon completion of the erosion duration of 90 days, the sulfate concentration increased significantly, approximately doubling compared to earlier stages. However, in nano-SiO_2_-modified RCA60 concrete, the sulfate concentration decreased by approximately half compared to unmodified RAC. This can be attributed to several factors. Firstly, the old mortar attached to the surface of RCA60 possesses a high density, which contributes to its resistance to sulfate penetration. Additionally, the filling effect of nano-SiO_2_ further enhances the density of the concrete matrix by filling internal pores. Secondly, the consumption of Ca(OH)_2_ during the modification process inhibits the formation of AFt and gypsum, which are known to cause expansion and degradation of RAC. Consequently, the combined effects of these factors result in a reduced sulfate concentration and enhanced durability of nano-SiO_2_-modified RAC compared to unmodified RAC. Overall, findings highlight the importance of modifications such as nano-SiO_2_ incorporation and carbonation treatment in enhancing the resistance of RAC to sulfate erosion. These modifications not only improve the compressive strength of RAC but also mitigate the adverse effects of sulfate penetration, thereby contributing to the overall durability and sustainability of RAC in construction applications.

### 3.3. Thermogravimetric Analysis

**Figure 6:**
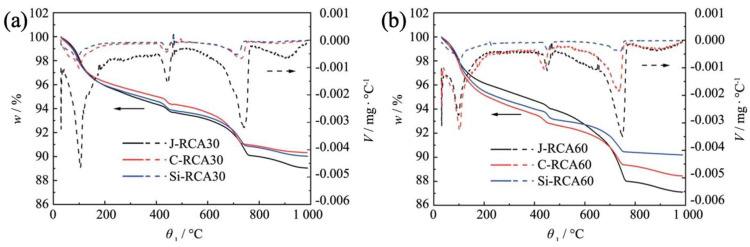
TGA curves of RAC: (**a**) RCA30 group; (**b**) RCA60 group.

Figure 6 delineates the findings derived from thermogravimetric analysis, offering insights into the compositional variations within modified and unmodified RAC. Upon the graphical representation, a discernible trend emerges: the mass fractions of unmodified, intermediate states, and CaCO_3_ are markedly elevated in the non-modified RAC vis-à-vis their counterparts in the modified variant. This observation underscores the efficacy of the modification process in mitigating sulfate attack and curbing the production of expansive ettringite (AFt) phases, as elucidated in the previous literature.

With respect to RCA60, which denotes recycled concrete aggregate incorporated at a 60% replacement level, the application of carbonation treatment failed to elicit a reduction in the mass fractions of sulfate-bearing phases. This anomaly can be rationalized by considering the dense, compact nature of high-strength RCA60 surfaces. Such density acts as a barrier, impeding the ingress of CO_2_ into the matrix, thereby hindering its interaction with the old mortar components [40]. Conversely, the incorporation of nano-silica particles introduced a marked decline in the mass fractions of the aforementioned sulfate-related compounds. This phenomenon attests to the remarkable capacity of nano-silica to infiltrate the RAC matrix, thereby refining its internal microstructure. This refinement, in turn, enhances the sulfate resistance of the modified RAC, fortifying its durability against aggressive sulfate environments.

### 3.4. Micro-Mechanical Characterization of RAC

**Figure 7:**
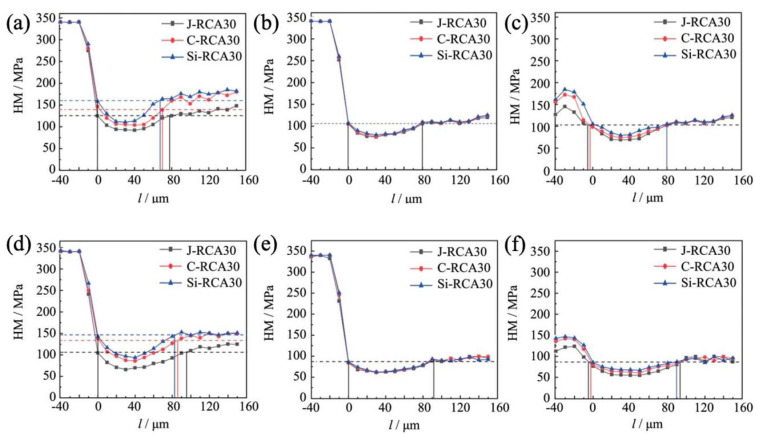
Micro-hardness of the ITZs of the RCA30 group: (**a**) LG-LS-ITZ-45d; (**b**) LG-XS-ITZ-45d; (**c**) LS-XS-ITZ-45d; (**d**) LG-LS-ITZ-90d; (**e**) LG-XS-ITZ-90d; (**f**) LS-XS-ITZ-90d.

The examination of the microhardness distribution profiles within the Interface Transition Zone (ITZ) of RCA30 and RCA60 recycled concretes offers profound insights into the mechanical characteristics and durability of these materials under sulfate erosion conditions. Figure 7 elucidates the intricate variations in microhardness within the ITZ of RCA30 recycled concrete, with a particular emphasis on the old aggregate-old mortar interface. This interface represents a critical zone where mechanical property disparities are most pronounced.

At an erosion duration of 45 days, the microhardness values within the LG-LS-ITZ (hereinafter referred to as the interface between the old aggregate and the old mortar in recycled concrete, for clarity) of J-RCA30 ranged between 91.4 and 125.8 MPa, accompanied by an interface zone width of approximately 76 μm. In a comparative analysis, C-RCA30 and Si-RCA30 recycled concrete exhibited microhardness enhancements of 14.0% to 9.5% and 21.7% to 28.9%, respectively, when benchmarked against J-RCA30. Concurrently, there was a notable reduction in interface zone widths by 10.8% and 13.5% for C-RCA30 and Si-RCA30, respectively. These observations suggest that modification processes can significantly influence the mechanical properties of the ITZ.

Upon extending the erosion period to 90 days, the microhardness values within the LG-LS-ITZ of J-RCA30 decreased to a range of 66.3 to 104.7 MPa, indicating a deterioration in mechanical properties. This decline was accompanied by an expansion in interface zone width to 98 mm, further highlighting the adverse effects of sulfate erosion. In stark contrast, C-RCA30 and Si-RCA30 recycled concretes demonstrated more resilient microhardness profiles, with increases ranging from 29.6% to 27.5% and 41.8% to 35.9%, respectively, compared to J-RCA30. Concurrently, the interface zone widths for these modified concretes decreased by 11.2% and 13.9%, respectively. These findings underscore the importance of material composition and treatment strategies in mitigating the detrimental effects of sulfate erosion on the mechanical properties of recycled concretes.

**Figure 8:**
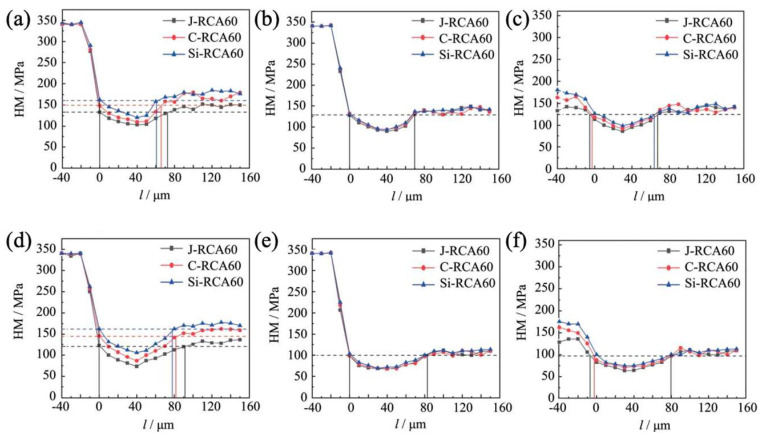
Micro-hardness of the ITZs of the RCA60 group: (**a**) LG-LS-ITZ-45d; (**b**) LG-XS-ITZ-45d; (**c**) LS-XS-ITZ-45d; (**d**) LG-LS-ITZ-90d; (**e**) LG-XS-ITZ-90d; (**f**) LS-XS-ITZ-90d.

Figure 8 presents a parallel analysis of the microhardness distribution within the ITZ of RCA60 recycled concrete. Similarly to RCA30, the old aggregate-old mortar interface emerged as a critical zone of mechanical vulnerability. At an erosion duration of 45 days, the microhardness values within the LG-LS-ITZ of J-RCA60 ranged from 102.3 to 131.6 MPa, with an interface zone width of approximately 74 μm. When compared to J-RCA60, C-RCA60, and Si-RCA60, recycled concretes exhibited microhardness enhancements of 6.6% to 12.3% and 15.5% to 21.7%, respectively. Concurrently, there was a reduction in interface zone widths by 8.9% and 14.9% for C-RCA60 and Si-RCA60, respectively.

Upon extending the erosion period to 90 days, the microhardness values within the LG-LS-ITZ of J-RCA60 decreased to a range of 74.5 to 119 MPa, accompanied by an expansion in interface zone width to 92 μm. In a comparative analysis, C-RCA60 and Si-RCA60 recycled concrete maintained more resilient microhardness profiles, with increases ranging from 6.7% to 10.8% and 15.5% to 21.2%, respectively, compared to J-RCA60. Concurrently, the interface zone widths for these modified concretes decreased by 11.5% and 13.8%, respectively. These observations suggest that, despite exhibiting similar trends in microhardness deterioration and interface zone expansion under sulfate erosion, the magnitude of these changes is notably smaller in RCA60 compared to RCA30. This finding underscores the enhanced durability and resistance to sulfate erosion exhibited by higher-strength RCA materials, thereby highlighting the importance of considering aggregate strength in assessing the long-term performance of recycled concretes in sulfate-laden environments.

### 3.5. Mechanism of the Modification Techniques Used to Strengthen RAC

Upon a synthesis of the aforementioned research endeavors, it becomes unequivocally apparent that both modification techniques are efficacious in refining the surface microstructure of RCA and augmenting the sulfate resistance of RAC. However, a comparative analysis reveals that, in contrast to modification through the incorporation of nano-SiO_2_, the enhancement imparted by carbonation is relatively modest, particularly in the context of lower-strength grade RCA30. The adherence of old mortar to the RCA surface introduces a pronounced interface barrier effect, which poses a significant challenge for fresh cement particles to effectively fill the voids in the vicinity of the aggregate surface. This phenomenon results in the formation of multiple interfaces enriched with structural imperfections. Carbonation treatment mitigates this issue by facilitating the reaction of Ca(OH)_2_ and C-S-H adhering to the RCA surface with CO_2_, yielding insoluble CaCO_3_ [41,42] (as delineated in the following equations).

$$\begin{array}{c} Ca{\left(OH\right)}_{2}+{CO}_{2}\to {CaCO}_{3}+H_{2}O\\ C-S-H+{CO}_{2}\to {CaCO}_{3}+{SiO_{2}\cdot xH}_{2}O \end{array}$$

This reaction serves to fill a portion of the pores and cracks on the RCA surface, as vividly illustrated in Figure 1b. Notably, RCA60, which exhibits a higher concentration of Ca(OH)_2_ adhering to its surface, undergoes more extensive carbonation, leading to the generation of a greater quantity of CaCO_3_. Consequently, the surface pores and cracks of RCA60 are markedly reduced, as depicted in the accompanying figures. Despite these positive outcomes, the reduction in C-S-H on the RCA surface subsequent to carbonation results in a decrement in interfacial bonding strength. The dissolution of calcium carbonate on the carbonated RCA surface upon contact with fresh cement paste releases CO_3_^2−^ ions, which react with aluminate ions within the cement paste to form C_3_A·CaCO_3_·11H_2_O. This process leads to the continuous erosion of CaCO_3_ on the RCA surface. While this erosion may contribute to an improvement in surface bonding, the uneven nature of these reactions results in the accumulation of defects at the interface. Furthermore, there exists a paradoxical relationship between the dissolution of CaCO_3_ and the nucleation of C-S-H on the surface. These intricate interfacial transformations are postulated to be the primary factors underlying the relatively modest enhancement effect observed with carbonation.

In stark contrast, the pozzolanic activity of nano-silica enables it to react with Ca(OH)_2_ adhering to the surface, thereby enhancing interfacial bonding [43]. Additionally, the nano-SiO_2_ adsorbed on the surface can disperse into the fresh cement paste, optimizing its internal microstructure and significantly elevating its mechanical properties and resistance to erosion. The incorporation of nano-SiO_2_ not only addresses the interface barrier effect but also fortifies the overall durability and performance of the recycled concrete, thereby presenting a more promising modification strategy compared to carbonation.

## 4. Conclusions

In conclusion, the novelty of this study lies in the effective utilization of both nano-silica soaking and carbonation techniques to enhance the sulfate resistance and compressive strength of recycled aggregate concrete derived from both normal and high-strength reference concretes, thereby offering new avenues for improving the durability and performance of sustainable concrete materials. The key findings are shown below.

- The porosity and crack density in the ITZ between the recycled aggregate and the cement paste in RAC are significant. Studies have shown that the porosity in the ITZ of RAC can be up to 30% higher than in virgin aggregate concrete. Sulfate ions readily penetrate through these pathways, leading to the formation of expansive ettringite, which causes deterioration in the mechanical properties of RAC. Under sulfate attack, RAC has been observed to lose up to 40% of its compressive strength.
- Carbonation treatment was found to be moderately effective in improving sulfate resistance. After carbonation, the sulfate penetration depth in RAC was reduced by approximately 15%. However, nano-silica incorporation demonstrated significantly greater efficacy. The incorporation of 2% nano-silica by weight of cement reduced the sulfate penetration depth by over 30% and the sulfate concentration by 25% compared to unmodified RAC. The compressive strength of nano-silica-modified RAC after 28 days of sulfate attack was increased by approximately 15% compared to the unmodified RAC.
- The sulfate ion mass equilibrium depth in nano-silica-modified RAC was reduced by approximately 30% compared to unmodified RAC. The sulfate concentration in the pore solution of nano-silica-modified RAC was also significantly lower, indicating improved sulfate resistance. After 56 days of sulfate attack, the compressive strength of nano-silica-modified RAC was maintained at 85% of its initial value, while that of unmodified RAC dropped to 70%.
- The quality of the recycled aggregate significantly influences the sulfate resistance of RAC. RCA derived from high-strength concrete (greater than 40 MPa) exhibited superior sulfate resistance compared to RCA from low-strength concrete (less than 20 MPa). The smoother and denser surface of high-strength RCA effectively mitigated sulfate ion ingress. Under sulfate attack, RAC made with high-strength RCA lost only 20% of its compressive strength, while RAC with low-strength RCA lost 40%.

The incorporation of nano-silica into RAC is a highly effective strategy to significantly enhance its sulfate resistance. This finding has important implications for the development of durable RAC formulations for use in sulfate-exposed environments. Future research should focus on optimizing the dosage of nano-silica and exploring other potential modifiers, such as mineral admixtures or fiber reinforcement, to further improve the durability of RAC. Additionally, the use of high-quality recycled aggregate should be prioritized in RAC production to maximize its sulfate resistance and overall performance. This can be achieved through improved aggregate processing techniques and stricter quality control measures. These findings provide valuable insights for the development of durable RAC formulations and contribute to the advancement of sustainable construction materials.

## Figures and Tables

**Table 1:** Mix proportion of the parent concrete (kg/m^3^).

Grade	Cement	FA	NCA	w/b	WR	f-28 d (MPa)
C30	300	800	1200	0.38	0.9	47.1
C60	530	680	1000	0.32	1.2	64.7

Note: FA—fine aggregate, NCA—natural coarse aggregates, w/b—water to binder ratio, WR—water reducer (polycarboxylate-based superplasticizer).

## Data Availability

The original contributions presented in this study are included in the article. Further inquiries can be directed to the corresponding authors.
